# Multiple clinical characteristics separate *MED12-*mutation-positive and -negative uterine leiomyomas

**DOI:** 10.1038/s41598-017-01199-0

**Published:** 2017-04-21

**Authors:** Hanna-Riikka Heinonen, Annukka Pasanen, Oskari Heikinheimo, Tomas Tanskanen, Kimmo Palin, Jaana Tolvanen, Pia Vahteristo, Jari Sjöberg, Esa Pitkänen, Ralf Bützow, Netta Mäkinen, Lauri A. Aaltonen

**Affiliations:** 1 Department of Medical and Clinical Genetics and Genome-Scale Biology Research Program, P.O. Box 63, FIN-00014 University of Helsinki, Helsinki, Finland; 2 Department of Pathology, P.O. Box 21, FIN-00014 University of Helsinki and Helsinki University Hospital, Helsinki, Finland; 3 Department of Obstetrics and Gynaecology, P.O. Box 140, FIN-00029 University of Helsinki and Helsinki University Hospital, Helsinki, Finland

## Abstract

Up to 86% of uterine leiomyomas harbour somatic mutations in *mediator complex subunit 12 (MED12)*. These mutations have been associated with conventional histology, smaller tumour size, and larger number of tumours within the uterus. Prior studies, with limited sample sizes, have failed to detect associations between other clinical features and *MED12* mutations. Here, we prospectively collected 763 uterine leiomyomas and the corresponding normal myometrial tissue from 244 hysterectomy patients, recorded tumour characteristics, collected clinical data from medical records, and screened the tissue samples for *MED12* mutations to assess potential associations between clinical variables and mutation status. Out of 763 leiomyomas, 599 (79%) harboured a *MED12* mutation. In the analysis of tumour characteristics, positive *MED12-*mutation status was significantly associated with smaller tumour size, conventional histology, and subserous location, relative to intramural. In the analysis of clinical variables, the number of *MED12-*mutation-positive tumours showed an inverse association with parity, and the number of mutation-negative tumours showed a positive association with a history of pelvic inflammatory disease. This study confirmed the previously reported differences and discovered novel differentiating features for *MED12-*mutation-positive and -negative leiomyomas. These findings emphasise the relevance of specific driver mutations in genesis and presentation of uterine leiomyomas.

## Introduction

Uterine leiomyomas are steroid-hormone-dependent benign smooth muscle tumours with an overall 70 to 80% incidence by 50 years of age^1^. They cause significant morbidity and substantial cost^2^. Typically, an affected uterus contains multiple leiomyomas ranging from a few millimetres up to 30 centimetres in size^3^. These lesions are histologically benign, but some display malignant features; these represent rare histopathological variants, such as cellular or mitotically active leiomyomas^4^. Approximately 25% of women with leiomyomas display symptoms, which depend on the number, location, and size of the tumours^5^. Even small submucous leiomyomas can cause heavy and irregular menstrual bleeding, whereas large intramural or subserous lesions can lead to pelvic pain, pressure, and urinary distress. Medication such as tranexamic acid and hormone therapy (HT) may relieve the symptoms, however, with a limited effect. Many patients therefore undergo surgical treatment; leiomyomas are the primary indication for hysterectomy.

Well-established risk factors for uterine leiomyomas include African origin, increasing age up to menopause, early menarche, nulliparity, infertility, family history of leiomyomas, and rare tumour susceptibility syndromes, particularly hereditary leiomyomatosis and renal cell cancer (HLRCC)^1, 6–12^. Obesity, alcohol intake, HT, reproductive tract infections, hypertension, and hypothyroidism also seem to cause increased risk for leiomyomas, whereas smoking and diabetes reduce their incidence^6, 8, 10, 11, 13–15^. Oral contraceptive users may be at reduced risk^6, 10^, although contradicting results exist^9^.

Up to 86% of uterine leiomyomas harbour site-specific mutations in *mediator complex subunit 12* (*MED12*)^16–27^. Based on gene expression profiling, leiomyomas carrying these mutations represent a molecularly distinct subtype^28^. Furthermore, *MED12* mutations are associated with smaller tumour size and larger number of tumours within the uterus, and they are less frequent in histopathological leiomyoma variants^16, 17, 20–23, 25, 27^. Earlier studies, with limited sample sizes, have failed to detect underlying associations between other clinical factors and *MED12*-mutation status, however^17, 18, 20, 22, 24^. To scrutinise these associations further, we have prospectively collected a series of 763 leiomyomas from 244 hysterectomy patients with comprehensive clinical data. These samples underwent screening for *MED12*-hotspot mutations, after which we tested the association of the mutation status with tumour characteristics and clinical factors.

## Results

**Figure 1:**
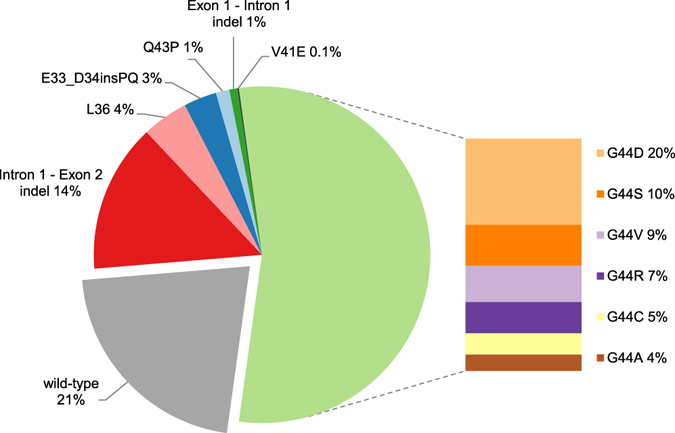
*MED12*-mutation spectrum in 763 uterine leiomyomas. In addition to negative mutation status (wild-type), missense mutations affecting different codons, and in-frame insertion and/or deletions (indel) in exon 2 or in intron 1- exon 2 junction and in exon 1 or exon 1 - intron 1 junction are shown separately. Codon 44 mutations are represented in more detail.

Out of 244 patients, 177 (73%) had at least one *MED12-*mutation-positive leiomyoma, and out of 763 uterine leiomyomas, 6 (1%) harboured a mutation in exon 1 and 593 (78%) in exon 2 (see Supplementary Table S1 for more detailed information). Table 1 presents the frequencies of *MED12* mutations for different leiomyoma subgroups. The majority of mutations, observed in 415 (54%) leiomyomas, were missense mutations affecting codon 44 (Fig. 1). All the mutations were heterozygous and somatic.

**Table 1:** *MED12* mutations in leiomyoma subgroups.

Subgroup	*MED12-*mutation positive n (%)	N
All leiomyomas	599 (78.5)	763
Leiomyomas from		
Finns	535 (78.8)	679
non-Finns	64 (76.2)	84
Histotype		
conventional leiomyoma	589 (79.7)	739
cellular leiomyoma	4 (40.0)	10
highly cellular leiomyoma	1 (25.0)	4
mitotically active leiomyoma	1 (50.0)	2
cellular and mitotically active leiomyoma	2 (100.0)	2
leiomyoma with bizarre nuclei	1 (50.0)	2
epithelioid leiomyoma	1 (100)	1
lipoleiomyoma	0 (0)	3

The median number of leiomyomas was 2 (range 1–16), the median number of *MED12*-mutation-positive tumours was 2 (range 0–16), and the median number of mutation-negative tumours was 1 (range 0–5) per patient. The number of *MED12-*mutation-positive and -negative leiomyomas were negatively correlated [Spearman’s correlation coefficient −0.52, P value (*P*)  = 1.6 × 10^−18^]. In addition, the number of *MED12-*mutation-positive tumours and the total number of leiomyomas were strongly correlated (Spearman’s correlation coefficient 0.87, *P* = 1.3 × 10^−77^), whereas the number of mutation-negative tumours and total number of leiomyomas showed no correlation.

First, to test whether tumour size, location and histotype are associated with the *MED12*-mutation status of the leiomyomas, we fit a generalised estimating equations (GEE) model. Positive mutation status was significantly associated with smaller tumour size (*P* = 4.2 × 10^−9^), conventional histology (*P* = 0.0013), and subserous relative to intramural location (*P* = 0.00082, Table 2).

**Table 2:** Leiomyoma characteristics and results of the generalised estimating equations model including leiomyoma size, location and histotype (n = 683).

Leiomyoma characteristics	All leiomyomas (N = 763)	*MED12-*mutation-positive (n = 599)	*MED12-*mutation-negative (n = 164)	Multivariable-adjusted regression coefficient [95% CI]	*P*
Size (cm)^1^	3.0 (2.0–5.0)	3.0 (1.7–4.0)	4.0 (2.2–7.0)	−0.18 [−0.24, −0.12]	**4.2 × 10** ^**−9**^
Histopathological variant	24 (3.3)	10 (1.7)	14 (8.5)	−1.60 [−2.58, −0.63]	**0.0013**
Location^2^					
intramural	559 (73.2)	419 (69.9)	140 (85.4)	reference	1
subserous	88 (11.6)	80 (13.4)	8 (4.9)	0.82 [0.34, 1.30]	**0.00082**
submucous	44 (5.8)	38 (6.3)	6 (3.7)	0.52 [−0.12, 1.16]	0.11

Data are median (inter quartile range) or n (%) unless otherwise specified. To adjust for multiple comparisons, *P* < 0.00147 indicated statistical significance (the Bonferroni correction). CI = confidence interval. ^1^15 missing values: 13 mutation-positive and 2 –negative. ^2^72 missing values: 62 mutation-positive and 10 –negative.

Second, we examined the associations between clinical factors and the number of *MED12-*mutation-positive leiomyomas. Here, we used negative binomial regression to account for overdispersion in the corresponding Poisson model (overdispersion test *P* = 7.1 × 10^−6^). The number of mutation-positive tumours was inversely associated with parity (*P* = 0.00017, Table 3). In addition, postmenopausal women without HT had fewer mutation-positive tumours, in comparison to premenopausal women; however, after adjusting for multiple testing, the outcome was not statistically significant (*P* = 0.0080). No associations were observed between the number of mutation-positive leiomyomas and age at hysterectomy, infertility, smoking status, body mass index (BMI), family history of leiomyomas, oral contraceptive use, history of pelvic inflammatory disease (PID) and chlamydia, hypertension, thyroid disorder, diabetes, or prior leiomyoma surgery.

**Table 3:** Characteristics of uterine leiomyoma patients and results from negative binomial and Poisson models for *MED12*-mutation-positive and –negative leiomyomas, respectively.

Patient characteristics	Median (IQR)/n (%) N = 244	NB model for number of *MED12*-mutation-positive leiomyomas (n = 241)		Poisson model for number of *MED12*-mutation-negative leiomyomas (n = 241)	
		**Multivariable-adjusted regression coefficient [95% CI]**	***P***	**Multivariable-adjusted regression coefficient [95% CI]**	***P***
Age at hysterectomy (y)	49 (45–54)	0.017 [−0.011, 0.046]	0.22	0.020 [−0.0099, 0.050]	0.19
Menopausal status					
premenopausal	178 (73.0)	reference	1	reference	1
current use of HT	33 (13.5)	−0.16 [−0.67, 0.36]	0.54	−0.41 [−1.01, 0.15]	0.17
postmenopausal, no HT	33 (13.5)	−0.85 [−1.49, −0.22]	0.0080	−0.49 [−1.18, 0.16]	0.15
Parity	2 (0–2)	−0.23 [−0.35, −0.10]	**0.00017**	0.069 [−0.054, 0.19]	0.26
Infertility	22 (9.0)	0.092 [−0.38, 0.59]	0.71	−0.18 [−0.83, 0.37]	0.54
Use of oral contraceptives (never/ever)	122 (50.0)	−0.040 [−0.34, 0.26]	0.78	0.015 [−0.31, 0.34]	0.93
History of PID	11 (4.5)	−0.17 [−0.87, 0.57]	0.63	1.05 [0.46, 1.59]	**0.00024**
History of chlamydia	10 (4.1)	−0.75 [−1.58, 0.075]	0.076	−0.12 [−0.97, 0.58]	0.75
Prior myomectomy	24 (9.8)	0.41 [−0.045, 0.88]	0.075	−0.41 [−1.14, 0.20]	0.22
Family history of leiomyomas	38 (15.6)	0.32 [−0.051, 0.71]	0.091	0.061 [−0.39, 0.47]	0.78
Hypertension	59 (24.2)	0.024 [−0.35, 0.40]	0.90	0.20 [−0.19, 0.58]	0.31
Diabetes mellitus	16 (6.6)	−0.13 [−0.75, 0.51]	0.69	0.29 [−0.36, 0.87]	0.35
Thyroid disease	24 (9.8)	−0.21 [−0.70, 0.30]	0.40	−0.16 [−0.78, 0.39]	0.60
Body mass index (kg/m^2^)^1^	25.6 (23.1–29.8)	0.0052 [−0.027, 0.037]	0.74	0.0090 [−0.025, 0.043]	0.60
Smoker (never/ever)^2^	63 (26.1)	0.17 [−0.15, 0.49]	0.30	−0.016 [−0.38, 0.33]	0.93

To adjust for multiple comparisons, *P* < 0.00147 indicated statistical significance (the Bonferroni correction). CI = confidence interval; IQR = inter quartile range; HT = hormone therapy; NB = negative binomial; PID = pelvic inflammatory disease. ^1^One value missing. ^2^Three values missing.

Finally, we assessed the associations between the clinical factors and the number of *MED12-*mutation-negative leiomyomas with Poisson regression. The number of mutation-negative tumours showed a statistically significant association with a history of PID (*P* = 0.00024, Table 3). No associations were observed between the number of mutation-negative leiomyomas and any of the other above-mentioned clinical factors.

## Discussion

This study examined the associations between *MED12*-mutation status, tumour characteristics, and various clinical factors in a large sample set of leiomyomas. As novel findings, the *MED12*-mutation-positive tumours were associated with subserous type and their number was inversely associated with parity, whereas the number of mutation-negative tumours was associated with a history of PID. In addition, our data confirmed the previous findings that *MED12*-mutation-positive leiomyomas are smaller in size and more often histopathologically conventional than mutation-negative lesions^17, 20–23, 25, 27^.

In this prospectively collected leiomyoma series, the frequency of *MED12* mutations reached 79%, being among the highest ones reported. Both Finns and patients of non-Finnish origin harboured mutations with similar frequencies. In prior studies with varying sample sizes and patient ethnicities, the mutation frequency in conventional leiomyomas has ranged from 31 to 86%, and in Finns, from 70 to 86%^16–18, 20–27^. The majority of the prior studies have only screened for exon 2 mutations, however. Here, 1% of leiomyomas harboured a mutation in exon 1, which is in line with the literature where the prevalence has ranged from 1 to 2%^17, 19^. Thus, exclusion of exon 1 mutations explains only a minor proportion of the variation seen in the reported mutation frequencies. One likely contributing factor explaining the different results is size bias: in some sample sets large lesions may have been preferred; this selects against *MED12*-mutation-positive tumours, ones usually smaller in size.

As expected, histopathological leiomyoma variants tended to be *MED12-*mutation negative. Nevertheless, 42% of the variants harboured a *MED12* mutation: a higher frequency than in previous works with mutation frequencies ranging from 0 to 21%^21, 23, 25, 27^. Due to the small number of leiomyoma variants in this study, however, it is difficult to draw definitive conclusions on their exact mutation frequencies. All three of the lipoleiomyomas were mutation negative. Only one prior study has screened *MED12* mutations from lipoleiomyomas, and similarly all four of the studied lesions were mutation negative^21^. Overall, our results are compatible with the previous notion that mechanisms other than *MED12* mutations, such as *high mobility group AT-hook 2* (*HMGA2*) aberrations, drive the majority of histopathological variants while the great majority of common leiomyomas are *MED12*-mutation positive^23, 29–32^.

This is, to our knowledge, the first study to show that positive *MED12-*mutation status is associated with subserosal location of leiomyomas. Two prior studies have explored the association between *MED12-*mutation status and tumour location with negative results, possibly due to different study settings and smaller sample sizes^18, 24^. Brosens *et al*. have concluded that submucosal leiomyomas display significantly fewer clonal cytogenetic changes compared to the other types^33^. Myometrium consists of functionally and structurally distinct layers: the subendometrial myometrium exhibits a cyclic pattern of steroid hormone receptor expression, whereas the outer layers of the uterine wall express these receptors continually^34^. Taken together, these data suggest that the differences in the myometrial layers influence the molecular mechanisms underlying leiomyoma development.

In addition, our results confirmed the previous findings that smaller tumour size and larger number of tumours are associated with positive *MED12*-mutation status^17, 20, 22, 24^. The small size of mutation-positive lesions may result from the coexistence of multiple tumours, which jointly lead to clinical intervention sooner, whereas the solitary, typically intramural, mutation-negative tumours need to grow larger to manifest symptoms. Alternatively, the biological growth process may differ between these leiomyoma subtypes. The multiplicity of *MED12-*mutation-positive leiomyomas may derive from genetic predisposition and/or environmental factors rendering the myometrium susceptible to selection for *MED12* mutations.

Intriguingly, a history of PID was associated with the number of *MED12-*mutation-negative tumours, whereas chlamydia infection, which can overlap with PID diagnosis, showed no association. A few epidemiological studies have observed an association between reproductive tract infections and increased risk for leiomyomas^13, 14^. Faerstein *et al*. observed increased odds for leiomyomas with a history of PID and chlamydia infection, as well as with past intrauterine device use when related to infectious complications, suggesting that inflammation may contribute to increased risk^13^. Furthermore, Moore *et al*. observed a negative association between chlamydia infection and multiple leiomyomas, and a similar trend for PID^14^, which is consistent with our result as mutation-negative leiomyomas are typically solitary. In the context of mutation-negative lesions, this finding is compatible with a proposed hypothesis that leiomyomas can occasionally develop via abnormal response to tissue repair or inflammatory stimuli, leading to cell proliferation and fibrosis^35^. It is also possible that an infectious agent may have a direct tumourigenic effect, such as the Epstein-Barr virus, which is known to cause leiomyomas and leiomyosarcomas in immunosuppressed patients^36^.

Evidently, parity reduces the risk of leiomyomas, with risk declining as the number of births increases^9, 10^. According to one proposed mechanism, remodelling of the uterus after each pregnancy would induce leiomyoma regression; however, direct evidence is lacking^37^. The inverse association between the number of *MED12-*mutation-positive leiomyomas and parity suggests that births reduce the risk specifically for this leiomyoma subgroup. As Baird and Dunson reason, uterine involution may be more potent in eliminating small lesions, ones typically mutation-positive, whereas remnants of larger tumours would survive^37^. In addition, the mutation-positive and -negative tumours may respond differently to the changes in, for example, the hormonal environment during and after pregnancy. Furthermore, postmenopausal women without HT showed a trend towards fewer *MED12-*mutation-positive leiomyomas than premenopausal women; however, we observed a similar, yet weaker, trend also with mutation-negative tumours, but due to the limited sample size no conclusions can be drawn. It is conceivable that *MED12-*mutation-positive and -negative leiomyomas may respond differently to hormone-level changes, as well as to treatments, such as selective progesterone receptor modulators.

The strength of this study is the large prospective sample set with comprehensive clinical data. On the other hand, the main shortcoming is the basic problem of any database survey; misclassification of variables is possible because of missing information. In addition, this study pooled all the *MED12-*mutation-negative leiomyomas into one group, although these lesions are likely to be heterogeneous due to the variety of the underlying genetic drivers^38, 39^. These include *HMGA2* rearrangements and biallelic inactivation of *fumarate hydratase* (*FH*), sometimes related to a germline mutation^30^. These two driver events and *MED12* mutations seem to be mutually exclusive and lead to unique gene expression patterns^28^. It is conceivable that all three leiomyoma subtypes may differ also in view of clinical characters, as it is already established that HLRCC-related FH-deficient tumours often show atypical histological features, develop at an earlier age, and are more numerous^7^, while HMGA2–aberrant tumours tend to be larger than other leiomyomas^40^. It remains to be elucidated, how the associations identified in this study relate to all three subgroups. Answering this question would require even larger sample sets due to the rarity of subtypes other than *MED12*, as well as much more extensive molecular workup to adequately identify the *FH* and *HMGA2* subgroups.

Clinical and molecular classification of uterine leiomyomas is likely to be important for developing targeted management strategies such as more effective drugs. This study confirmed the association of *MED12* mutations with smaller leiomyoma size, multiplicity and conventional histotype, and revealed novel associations between the *MED12*-mutation status and leiomyoma location, PID, and parity. These findings emphasise the relevance of specific driver mutations in genesis and presentation of uterine leiomyomas.

## Methods

### Subjects

This study was approved by the appropriate Ethics Review Board of the Helsinki University Hospital (HUH), Finland, and conducted in accordance with the Declaration of Helsinki. All patients gave signed informed consents before entering the study. We collected uterine leiomyoma (n = 763) and corresponding normal myometrial tissue samples from patients (n = 244) who had ultrasound-diagnosed uterine leiomyomas and underwent hysterectomy in HUH during October 2013 and November 2015 for any medical indication. Pathologists (A.P. and R.B.) dissected the hysterectomy specimens and recorded the location and size (the largest diameter) of the tumours. All feasible distinct tumours ≥1 cm in diameter and a piece of the corresponding normal myometrial tissue were harvested and stored as fresh frozen. In addition, a formalin-fixed paraffin-embedded (FFPE) block from each tumour was prepared. The dissected hysterectomy specimens underwent routine diagnostic pathological scrutiny, and the pathology reports provided the histopathological diagnosis for the study samples. A gynaecological pathologist (R.B.) reviewed haematoxylin-eosin-stained FFPE-tissue sections of all suspected (based on the pathology reports) leiomyoma variants and classified them according to the WHO 2014 criteria^4^.

Clinical data came from a retrospective review of medical records of all study subjects, and for 70 patients, from an additional designed questionnaire. Data included demographic information and medical and gynaecological history (Table 3).

### Mutation analysis

All tumour and normal tissue samples underwent genomic DNA extraction with FastDNA Kit (MP Biomedicals LLC, Solon, OH, USA) and screening for *MED12* exon 1 and 2 mutations by Sanger sequencing using 5′ to 3′ primers CCTCCGGAACGTTTCATAGAT (forward) and TTCGGGACTTTTGCTCTCAC (reverse), and GCCCTTTCACCTTGTTCCTT (forward) and TGTCCCTATAAGTCTTCCCAACC (reverse), respectively. PCR products were sequenced using Big Dye Terminator v.3.1 sequencing chemistry (Applied Biosystems, Foster City, CA, USA) on an ABI3730 Automatic DNA Sequencer. We analysed sequence graphs manually and with Mutation Surveyor software (Softgenetics, State College, PA, USA).

### Statistical methods

We employed R version 3.2.3 to perform statistical analyses^41^. Continuous variables were summarised as medians and ranges or inter quartile ranges (IQRs) due to non-normal distribution, and categorical variables as counts and percentages (Tables 2 and 3). We applied GEE, as well as Poisson and negative binomial regression, to obtain multivariable-adjusted effect size estimates (regression coefficients) accompanied by 95% confidence intervals (see Supplementary Information online for detailed information on the statistical models). In tumour-level analysis, *MED12*-mutation status (a binary variable) was predicted by tumour characteristics (size, location and histotype). Considering that multiple leiomyomas within a uterus may not be independent, we applied the GEE method with exchangeable correlation structure (80 observations deleted due to missing information, n = 683). The model was fit assuming the binomial random component and logit link function (geeglm function in R package geepack)^42–44^. Inferences of the GEE parameters were based on the Wald statistic. To model *MED12*-mutation-negative-leiomyoma counts, we applied Poisson regression (glm function in R, three observations deleted due to missing information, n = 241). In the model for *MED12*-mutation-positive leiomyoma counts, we assumed the negative binomial distribution with quadratic variance function (glm.nb function in R package MASS, three observations deleted due to missing information, n = 241) to account for overdispersion in the corresponding Poisson model (evaluated using dispersiontest function in R package AER)^45, 46^. To assess whether the negative binomial distribution provided a better fit to these data, we used the likelihood ratio test to compare negative binomial and Poisson regression models (odTest function in R package pscl)^47^. The generalised Pearson statistic and model deviance were used to assess Poisson and negative binomial goodness of fit. In both models for tumour counts, we considered explanatory variables previously associated with leiomyoma risk and putative confounding variables [African origin, age at hysterectomy, menarche, menopausal status (premenopausal/current use of HT/postmenopausal, no HT), parity, infertility, smoking status, alcohol use, BMI, family history of leiomyomas, oral contraceptive use, history of PID and chlamydia, hypertension, thyroid disorder, diabetes, and prior leiomyoma surgery]. Variables with >10 missing values (menarche and alcohol use) or <5 cases (African origin) were omitted. Spearman correlation matrix (R package PerformanceAnalytics) and variance inflation factors (vif function in R package car) were computed to evaluate possible collinearity among explanatory variables^48, 49^. A total of 34 statistical tests were undertaken to investigate the relationships between patient- or tumour-level variables and *MED12* mutations. To adjust for multiple comparisons, we applied the Bonferroni correction (α = 0.05/34); two-sided *P* < 0.00147 indicated statistical significance.

## Electronic supplementary material

Supplementary Information
