# Supplementary material for: Multiple clinical characteristics separate MED12-mutation-positive and -negative uterine leiomyomas
Source: Sci Rep. 2017 Apr 21;7:1015. doi: 10.1038/s41598-017-01199-0 (PMC5430741; doi:10.1038/s41598-017-01199-0)
Supplement: Supplementary file 1 — Supplementary Information [file 41598_2017_1199_MOESM1_ESM.pdf]

Multiple clinical characteristics separate MED12-mutation-positive and -negative uterine leiomyomas

Supplementary Information

Hanna-Riikka Heinonen, Annukka Pasanen, Oskari Heikinheimo, Tomas Tanskanen, Kimmo Palin, Jaana Tolvanen, Pia Vahteristo, Jari Sjöberg, Esa Pitkänen, Ralf Bützow, Netta Mäkinen, and Lauri A. Aaltonen

Statistical analysis

Below we display detailed information for the statistical models. To test whether tumour characteristics associate with the *MED12*-mutation status of the leiomyomas, we fit a generalized estimating equations (GEE) model. In the model for *MED12*-mutation-positive leiomyoma counts, we assumed the negative binomial distribution to account for overdispersion in the corresponding Poisson model (dispersiontest $P = 7.1 * 10^{-6}$). The negative binomial distribution fit the observed counts significantly better than the Poisson distribution (likelihood ratio test $P < 2.2 * 10^{-16}$). This negative binomial model satisfied the Pearson goodness of fit (GOF) test ($P = 0.16$), although the deviance GOF statistic remained significant ($P = 0.036$). In the model for *MED12*-mutation-negative-leiomyoma counts, we used Poisson regression. This model satisfied the overdispersion, Pearson and deviance GOF tests ($P = 0.87$, $P = 0.68$ and $P = 0.37$, respectively).

GEE model for the *MED12*-mutation status of the leiomyomas

```
##
## Call:
## geeglm(formula = `MED12-mutation status` ~ `Size (cm)` + `Location ` +
##     `Histopathological variant`, family = binomial(link = logit),
##     data = Tumour_data, id = `Patient ID`, corstr = "exchangeable")
##
##  Coefficients:
##                             Estimate  Std.err   Wald Pr(>|W|)
## (Intercept)                  1.61364  0.18767 73.927  < 2e-16 ***
## `Size (cm)`                 -0.18192  0.03096 34.533 4.19e-09 ***
## `Location `submucous         0.51986  0.32529  2.554 0.110015
## `Location `subserous         0.82092  0.24538 11.193 0.000821 ***
## `Histopathological variant` -1.60323  0.49694 10.408 0.001255 **
## ---
## Signif. codes:  0 '***' 0.001 '**' 0.01 '*' 0.05 '.' 0.1 ' ' 1
##
## Estimated Scale Parameters:
##             Estimate Std.err
## (Intercept)   0.8647  0.1256
##
## Correlation: Structure = exchangeable  Link = identity
##
## Estimated Correlation Parameters:
##       Estimate Std.err
## alpha    0.306 0.07826
## Number of clusters:   234   Maximum cluster size: 15
```

Poisson model for *MED12*-mutation-positive tumours

```
##
## Call:
## glm(formula = `Number of MED12-mutation-positive tumours` ~ `Age at hysterectomy (y)` +
##     BMI + `Mp status` + Parity + `History of chlamydia` + `History of PID` +
##     Infertility + `Family history of leiomyomas` + Hypertension +
##     Smoker + `Thyroid disease` + `Prior myomectomy` + `Diabetes mellitus` +
##     `Use of oral contraceptives`, family = poisson, data = Patient_data)
##
```

```
## Deviance Residuals:
##     Min       1Q   Median       3Q      Max
## -3.132  -1.544  -0.524   0.666   5.644
##
## Coefficients:
##                                   Estimate Std. Error z value Pr(>|z|)
## (Intercept)                        0.51746    0.44465    1.16  0.24452
## `Age at hysterectomy (y)`          0.00966    0.00804    1.20  0.22990
## BMI                                0.00778    0.00905    0.86  0.38982
## `Mp status`current use of HT      -0.12262    0.15148   -0.81  0.41825
## `Mp status`postmenopausal, no HT -0.77570    0.20688   -3.75  0.00018 ***
## Parity                            -0.20483    0.03634   -5.64  1.7e-08 ***
## `History of chlamydia`            -0.70180    0.28909   -2.43  0.01520 *
## `History of PID`                  -0.17917    0.21325   -0.84  0.40079
## Infertility                        0.02367    0.13812    0.17  0.86392
## `Family history of leiomyomas`     0.32403    0.10310    3.14  0.00167 **
## Hypertension                       0.07119    0.11366    0.63  0.53109
## Smoker                             0.07195    0.09479    0.76  0.44783
## `Thyroid disease`                 -0.19899    0.14665   -1.36  0.17481
## `Prior myomectomy`                 0.41065    0.11807    3.48  0.00050 ***
## `Diabetes mellitus`               -0.17692    0.19394   -0.91  0.36165
## `Use of oral contraceptives`      -0.05534    0.08574   -0.65  0.51864
## ---
## Signif. codes:  0 '***' 0.001 '**' 0.01 '*' 0.05 '.' 0.1 ' ' 1
##
## (Dispersion parameter for poisson family taken to be 1)
##
##     Null deviance: 735.16  on 240  degrees of freedom
## Residual deviance: 637.44  on 225  degrees of freedom
##   (3 observations deleted due to missingness)
## AIC: 1165
##
## Number of Fisher Scoring iterations: 5
```

Overdispersion test for the Poisson model

```
##
##  Overdispersion test
##
## data:  Poisson_model_for_MED12_mutation_positive_tumours
## z = 4.3, p-value = 7e-06
## alternative hypothesis: true alpha is greater than 0
## sample estimates:
##  alpha
## 0.7007
## [1] 7.145e-06
```

Negative binomial model for *MED12*-mutation-positive tumours

```
##
## Call:
## glm.nb(formula = `Number of MED12-mutation-positive tumours` ~
##     `Age at hysterectomy (y)` + BMI + `Mp status` + Parity +
##         `History of chlamydia` + `History of PID` + Infertility +
##         `Family history of leiomyomas` + Hypertension + Smoker +
##         `Thyroid disease` + `Prior myomectomy` + `Diabetes mellitus` +
##         `Use of oral contraceptives`, data = Patient_data, init.theta = 1.343197684,
##     link = log)
##
## Deviance Residuals:
##    Min      1Q  Median      3Q     Max
## -2.022  -1.262  -0.315   0.412   2.942
##
## Coefficients:
##                                  Estimate Std. Error z value Pr(>|z|)
## (Intercept)                       0.22669    0.76073    0.30  0.76571
## `Age at hysterectomy (y)`         0.01707    0.01385    1.23  0.21758
## BMI                               0.00523    0.01567    0.33  0.73847
## `Mp status`current use of HT     -0.15869    0.25694   -0.62  0.53683
## `Mp status`postmenopausal, no HT -0.85095    0.32071   -2.65  0.00797 **
## Parity                           -0.22501    0.05991   -3.76  0.00017 ***
## `History of chlamydia`           -0.74679    0.42072   -1.78  0.07589 .
## `History of PID`                 -0.16913    0.35470   -0.48  0.63350
## Infertility                       0.09188    0.24638    0.37  0.70922
## `Family history of leiomyomas`    0.32267    0.19101    1.69  0.09116 .
## Hypertension                      0.02404    0.19062    0.13  0.89965
## Smoker                            0.16864    0.16288    1.04  0.30051
## `Thyroid disease`                -0.20997    0.24845   -0.85  0.39804
## `Prior myomectomy`                0.40697    0.22829    1.78  0.07464 .
## `Diabetes mellitus`              -0.12694    0.31540   -0.40  0.68735
## `Use of oral contraceptives`     -0.04044    0.14757   -0.27  0.78405
## ---
## Signif. codes:  0 '***' 0.001 '**' 0.01 '*' 0.05 '.' 0.1 ' ' 1
##
## (Dispersion parameter for Negative Binomial(1.343) family taken to be 1)
##
##     Null deviance: 300.92  on 240  degrees of freedom
## Residual deviance: 264.73  on 225  degrees of freedom
##   (3 observations deleted due to missingness)
## AIC: 1003
##
## Number of Fisher Scoring iterations: 1
##
##
##               Theta:  1.343
##           Std. Err.:  0.216
##
##  2 x log-likelihood:  -968.667
```

Likelihood ratio test to compare Poisson and negative binomial models

```
## Likelihood ratio test of H0: Poisson, as restricted NB model:
## n.b., the distribution of the test-statistic under H0 is non-standard
## e.g., see help(odTest) for details/references
##
## Critical value of test statistic at the alpha= 0.05 level: 2.7055
## Chi-Square Test Statistic =  164.4427 p-value = < 2.2e-16
```

Pearson GOF test p-value for the negative binomial model

```
## [1] 0.1624
```

Deviance GOF test p-value for the negative binomial model

```
## [1] 0.03562
```

Poisson model for *MED12*-mutation-negative tumours

```
##
## Call:
## glm(formula = `Number of MED12-mutation-negative tumours` ~ `Age at hysterectomy (y)` +
##     BMI + `Mp status` + Parity + `History of chlamydia` + `History of PID` +
##     Infertility + `Family history of leiomyomas` + Hypertension +
##     Smoker + `Thyroid disease` + `Prior myomectomy` + `Diabetes mellitus` +
##     `Use of oral contraceptives`, family = poisson, data = Patient_data)
##
## Deviance Residuals:
##    Min      1Q  Median      3Q     Max
## -1.704  -1.091  -0.220   0.455   3.116
##
## Coefficients:
##                                   Estimate Std. Error z value Pr(>|z|)
## (Intercept)                       -1.74051    0.83910   -2.07  0.03805 *
## `Age at hysterectomy (y)`          0.02019    0.01534    1.32  0.18824
## BMI                                0.00901    0.01732    0.52  0.60287
## `Mp status`current use of HT      -0.41042    0.29578   -1.39  0.16526
## `Mp status`postmenopausal, no HT -0.49220    0.33987   -1.45  0.14756
## Parity                             0.06935    0.06201    1.12  0.26341
## `History of chlamydia`            -0.12377    0.39279   -0.32  0.75268
## `History of PID`                   1.05191    0.28612    3.68  0.00024 ***
## Infertility                       -0.18457    0.30401   -0.61  0.54378
## `Family history of leiomyomas`     0.06147    0.21934    0.28  0.77930
## Hypertension                       0.20163    0.19728    1.02  0.30677
## Smoker                            -0.01601    0.18090   -0.09  0.92947
## `Thyroid disease`                 -0.15598    0.29539   -0.53  0.59746
## `Prior myomectomy`                -0.41423    0.33767   -1.23  0.21993
## `Diabetes mellitus`                0.28946    0.31262    0.93  0.35450
```

```
## `Use of oral contraceptives`      0.01549    0.16694    0.09  0.92606
## ---
## Signif. codes:  0 '***' 0.001 '**' 0.01 '*' 0.05 '.' 0.1 ' ' 1
##
## (Dispersion parameter for poisson family taken to be 1)
##
##     Null deviance: 252.44  on 240  degrees of freedom
## Residual deviance: 231.19  on 225  degrees of freedom
##   (3 observations deleted due to missingness)
## AIC: 530.8
##
## Number of Fisher Scoring iterations: 5
```

Overdispersion test for the Poisson model

```
##
##  Overdispersion test
##
## data:  Poisson_model_for_MED12_mutation_negative_tumours
## z = -1.1, p-value = 0.9
## alternative hypothesis: true alpha is greater than 0
## sample estimates:
##   alpha
## -0.1587
## [1] 0.8713
```

Pearson GOF test p-value for the Poisson model

```
## [1] 0.678
```

Deviance GOF test p-value for the Poisson model

```
## [1] 0.3743
```

Supplementary Table S1. *MED12*-mutation status and tumour characteristics of 763 uterine leiomyomas from 244 patients.

Patient	Tumour	*MED12* status	Size (cm)	Type	Histopathological variant
My6002	T1	c.130G>A, p.G44S	5.0	NA	
	T2	c.130G>A, p.G44S	6.0	NA	
	T3	c.130G>C, p.G44R	4.0	NA	
My6004	T1	c.131G>A, p.G44D	8.5	intramural	
	T2	c.77_97del21, p.P26_E33delinsQ	3.5	subserous	
	T3	c.130G>A, p.G44S	2.0	subserous	
My6005	T1	c.128A>C, p.Q43P	1.8	intramural	
	T2	c.130G>A, p.G44S	1.2	intramural	
My6006	T1	wt	0.9	intramural	
My6008	T1	c.130G>A, p.G44S	7.0	intramural	
	T2	c.130G>C, p.G44R	5.0	intramural	
	T3	c.130G>C, p.G44R	3.0	intramural	
	T4	c.128A>C, p.Q43P	2.0	intramural	
My6009	T1	c.131G>A, p.G44D	4.5	intramural	
My6010	T1	c.130G>A, p.G44S	6.0	intramural	
	T2	c.130G>A, p.G44S	4.5	intramural	
	T3	wt	3.0	intramural	
My6011	T1	wt	8.0	intramural	
My6012	T1	wt	3.0	intramural	
	T3	wt	1.5	subserous	
My6013	T1	c.131G>T, p.G44V	1.8	intramural	
	T2	c.146_166del21, p.P49_E55del	1.8	intramural	
	T3	wt	2.2	intramural	
My6014	T1	c.131G>A, p.G44D	3.5	intramural	
	T2	c.131G>A, p.G44D	5.0	intramural	
My6015	T1	c.126_137del12, p.K42_F45del	6.0	intramural	
My6016	T1	c.131G>A, p.G44D	10.0	intramural	
	T2	c.130G>A, p.G44S	4.0	intramural	
My6017	T1	c.107T>G, p.L36R	7.0	intramural	
	T2	c.130G>C, p.G44R	6.0	intramural	
	T3	c.131G>T, p.G44V	5.0	intramural	
	T4	c.130G>C, p.G44R	3.0	intramural	
	T5	c.129_143del15, p.G44_Q48del	2.0	intramural	
My6018	T1	wt	7.0	intramural	
	T2	wt	4.0	intramural	
	T3	c.130G>C, p.G44R	4.0	intramural	
	T4	wt	2.5	intramural	
My6019	T1	c.131G>T, p.G44V	17.0	intramural	cellular and mitotically active leiomyoma
My6020	T1	c.131G>A, p.G44D	3.0	intramural	
	T2	c.131G>A, p.G44D	3.0	intramural	
	T3	wt	2.5	intramural	
My6021	T1	wt	2.0	intramural	
My6022	T1	wt	4.0	intramural	lipoleiomyoma
	T2	wt	2.0	intramural	
My6023	T1	wt	4.0	submucous	
My6024	T1	wt	10.0	NA	
My6025	T1	c.131G>A, p.G44D	13.0	intramural	
My6026	T1	c.131G>T, p.G44V	1.5	subserous	
My6027	T1	wt	3.0	intramural	
My6028	T1	c.131G>A, p.G44D	2.0	submucous	
My6030	T1	c.130G>A, p.G44S	8.0	subserous	

	T2	c.131G>A, p.G44D	3.0	intramural	
	T3	c.114_149del36, p.L39_A50del	2.5	intramural	
My6031	T1	c.131G>A, p.G44D	3.5	intramural	
	T2	c.131G>C, p.G44A	3.0	intramural	
	T3	c.100-3_132del36, loss of splice acceptor	3.0	submucous	
My6032	T1	c.131G>A, p.G44D	1.5	intramural	
My6033	T1	wt	11.0	intramural	
	T2	c.130G>A, p.G44S	4.0	intramural	
	T3	c.131G>A, p.G44D	3.5	intramural	
	T4	c.145_162del18insGAG, p.P49_D54delinsE	3.0	intramural	
My6034	T1	c.130G>T, p.G44C	4.0	intramural	
	T2	c.130G>C, p.G44R	2.0	submucous	
	T3	c.107T>G, p.L36R	1.5	intramural	
My6035	T1	c.130G>A, p.G44S	3.2	submucous	cellular leiomyoma
My6036	T1	c.130G>A, p.G44S	7.0	intramural	
	T2	c.126_137del12, p.K42_F45del	5.0	intramural	
	T3	c.131G>T, p.G44V	5.0	intramural	
	T4	c.118_153del36, p.N40_V51del	4.0	intramural	
	T5	wt	4.0	intramural	highly cellular leiomyoma
	T6	c.131G>T, p.G44V	3.0	intramural	
My6037	T1	wt	5.5	intramural	cellular leiomyoma
	T2	c.131G>T, p.G44V	2.0	intramural	
	T3	wt	2.2	intramural	
My6038	T1	wt	12.0	intramural	
My6039	T1	c.107T>G, p.L36R	ND	NA	
My6043	T1	c.131G>T, p.G44V	9.0	subserous	
	T2	c.144_167del24, p.Q48_E55del	8.0	intramural	
My6045	T1	c.131G>C, p.G44A	3.0	intramural	
My6046	T1	wt	8.0	intramural	
My6047	T1	c.131G>T, p.G44V	6.0	intramural	
	T2	c.131G>T, p.G44V	6.0	intramural	
	T3	c.130G>T, p.G44C	2.5	intramural	
	T4	c.131G>A, p.G44D	3.0	intramural	
	T5	c.131G>T, p.G44V	4.0	intramural	
	T6	wt	4.0	intramural	
My6048	T2	wt	2.5	intramural	
My6049	T1	c.131G>A, p.G44D	5.0	NA	cellular leiomyoma
	T2	c.131G>A, p.G44D	3.0	submucous	
	T3	c.130G>A, p.G44S	1.3	intramural	
	T4	c.107T>G, p.L36R	1.0	intramural	
My6050	T1	c.122_136del15, p.V41_N46delinsD	6.0	NA	
	T2	c.127_132delCAAGGT, p.Q43_G44del	7.0	subserous	
	T3	wt	6.0	intramural	
	T4	c.130G>A, p.G44S	5.0	intramural	
	T5	c.130G>A, p.G44S	3.5	intramural	
	T6	c.100-4_147del52, loss of splice acceptor	6.0	subserous	
	T7	c.131G>T, p.G44V	3.0	subserous	
	T8	c.123_131del9, p.K42_G44del	2.0	subserous	
	T9	c.131G>C, p.G44A	3.0	subserous	
	T10	c.131G>A, p.G44D	3.0	subserous	
My6051	T1	wt	ND	intramural	
My6052	T1	wt	1.8	intramural	
My6054	T1	c.131G>C, p.G44A	7.0	intramural	
	T2	c.107T>G, p.L36R	5.0	intramural	
	T3	c.131_160del30, p.G44_G53del	5.0	intramural	
	T4	c.131G>A, p.G44D	5.0	intramural	

	T5	c.105_134del30, p.E35_F45delinsD	3.5	intramural
	T6	c.131G>C, p.G44A	3.0	intramural
My6055	T1	c.131G>A, p.G44D	11.0	subserous
	T2	c.131G>A, p.G44D	4.0	subserous
	T3	c.131G>A, p.G44D	3.0	intramural
	T4	c.130G>C, p.G44R	2.5	intramural
My6056	T1	c.131G>T, p.G44V	4.5	intramural
My6057	T1	c.100-8T>A, p.E33_D34insPQ	6.0	intramural
	T2	c.131G>T, p.G44V	6.0	intramural
	T3	c.130G>A, p.G44S	ND	intramural
	T4	c.131G>A, p.G44D	3.0	intramural
	T5	c.[110_118dup9;119A>G], p.[T37_L39dup;N40S]	2.5	intramural
	T6	c.126_134del9, p.K42_F45delinsN	2.0	intramural
	T7	c.131G>A, p.G44D	1.5	NA
My6058	T1	c.130G>A, p.G44S	3.5	intramural
	T2	c.131G>A, p.G44D	2.6	intramural
	T3	c.130G>A, p.G44S	2.7	intramural
	T4	c.130G>T, p.G44C	1.0	submucous
My6059	T1	wt	6.0	intramural
My6061	T1	wt	10.5	intramural
My6062	T1	c.131G>A, p.G44D	2.0	intramural
My6063	T1	wt	9.5	intramural
	T2	c.131G>C, p.G44A	4.0	subserous
	T3	c.121_123delGTA, p.V41del	2.5	subserous
	T4	c.131G>T, p.G44V	2.0	intramural
My6064	T1	wt	9.0	intramural
My6065	T1	c.100-11_134del46, loss of splice acceptor	2.0	intramural
My6066	T1	wt	2.5	intramural
My6067	T1	wt	6.0	NA
	T2	c.141_155del15, p.Q48_S52del	3.0	intramural
My6068	T1	wt	12.0	intramural
My6069	T1	c.131G>A, p.G44D	3.0	submucous
My6070	T1	wt	5.0	NA
	T2	c.137_159del23insTA, p.N46_G53delinsI	2.0	intramural
My6071	T1	wt	4.5	intramural
My6072	T1	c.130G>T, p.G44C	3.0	NA
My6073	T1	wt	3.0	intramural
My6074	T1	c.131G>A, p.G44D	1.3	intramural
	T2	c.[107_142del36;149C>G], p.[L36_N47del;A50G]	1.5	intramural
	T3	c.131G>T, p.G44V	1.3	intramural
	T4	c.131G>A, p.G44D	1.5	intramural
My6075	T1	wt	6.0	intramural
My6076	T1	c.131G>T, p.G44V	11.0	intramural
	T2	c.131G>C, p.G44A	4.0	submucous
My6077	T1	c.121_138del18, p.V41_N46del	8.0	intramural
	T2	c.131G>A, p.G44D	4.0	intramural
	T3	c.100-1_139del41, loss of splice acceptor	ND	intramural
My6078	T1	wt	5.0	intramural
	T2	c.107T>G, p.L36R	3.0	intramural
	T3	wt	3.0	intramural
	T5	wt	1.5	intramural
My6080	T1	c.130G>T, p.G44C	6.0	intramural
	T2	wt	2.5	intramural
	T3	wt	1.5	intramural
	T4	c.100-8T>A, p.E33_D34insPQ	1.5	subserous

My6082	T1	wt	2.2	intramural
	T2	wt	2.5	submucous
My6083	T1	c.130G>T, p.G44C	1.4	intramural
My6084	T1	c.136_150del15, p.N46_A50del	5.0	intramural
	T2	c.107T>G, p.L36R	7.0	NA
	T3	c.131G>T, p.G44V	2.0	subserous
	T4	c.131G>T, p.G44V	4.0	NA
My6085	T1	wt	5.0	intramural
My6086	T1	c.131G>A, p.G44D	3.5	subserous
	T2	c.100-8T>A, p.E33_D34insPQ	1.5	intramural
	T3	c.130G>A, p.G44S	2.0	intramural
	T4	c.128_129insGTT, p.Q43_G44insL	1.0	intramural
My6087	T1	c.131G>A, p.G44D	6.0	intramural
	T2	c.131G>A, p.G44D	2.5	intramural
	T3	c.131G>A, p.G44D	2.0	intramural
	T4	c.131G>A, p.G44D	3.0	intramural
	T5	c.131G>A, p.G44D	4.0	NA
	T6	c.130G>C, p.G44R	1.5	submucous
My6088	T1	c.130G>C, p.G44R	4.5	intramural
My6089	T1	c.131G>A, p.G44D	2.5	intramural
	T2	c.130G>A, p.G44S	ND	subserous
	T3	wt	2.0	intramural
	T4	c.131G>A, p.G44D	1.5	intramural
My6090	T1	c.128_130delAAG, p.Q43_G44delinsR	7.0	intramural
	T2	c.131G>A, p.G44D	8.0	intramural
	T3	c.130G>A, p.G44S	5.0	intramural
My6091	T1	wt	12.0	intramural
My6092	T1	c.131G>T, p.G44V	5.0	intramural
	T2	c.131G>A, p.G44D	3.0	intramural
	T3	c.107_127del21, p.L36_K42del	3.5	intramural
	T4	c.101_121del21insCTGAAC, p.D34_V41delinsAEL	2.5	intramural
	T5	c.131G>A, p.G44D	3.0	intramural
	T6	c.131G>A, p.G44D	2.0	intramural
	T7	c.131G>T, p.G44V	3.0	submucous
	T8	c.131G>C, p.G44A	2.0	submucous
My6093	T1	wt	3.0	submucous
My6094	T1	c.131G>C, p.G44A	1.5	submucous
	T2	c.100-8T>A, p.E33_D34insPQ	1.0	subserous
My6095	T1	c.131G>T, p.G44V	4.0	NA
	T2	c.131G>A, p.G44D	4.0	NA
	T3	c.130G>A, p.G44S	3.0	NA
	T4	c.133_144del12, p.F45_Q48del	2.5	subserous
	T5	c.130G>A, p.G44S	1.5	intramural
	T6	c.107_112del6, p.L36_A38delinsP	1.5	intramural
My6096	T1	c.133_150del18, p.F45_A50del	4.0	NA
	T2	c.127_132del6, p.Q43_G44del	4.0	intramural
	T3	wt	1.5	intramural
My6097	T1	c.131G>A, p.G44D	4.0	subserous
	T2	c.131G>A, p.G44D	3.5	subserous
	T3	c.131G>T, p.G44V	4.5	subserous
	T4	c.131G>A, p.G44D	3.0	subserous
	T5	c.131G>T, p.G44V	3.5	subserous
	T6	c.130G>T, p.G44C	6.0	submucous
My6098	T1	wt	8.0	intramural
	T2	c.133_144del12, p.F45_Q48del	4.5	subserous
My6099	T1	wt	17.0	intramural

My6100	T1	wt	1.0	intramural	
My6101	T1	c.131G>A, p.G44D	4.0	intramural	
	T2	c.131G>A, p.G44D	2.5	intramural	
	T3	c.131G>T, p.G44V	2.0	intramural	
My6102	T1	wt	3.0	intramural	
	T2	c.125_133del9, p.K42_F45delinsI	0.8	intramural	
My6103	T1	c.131G>A, p.G44D	10.0	subserous	
	T2	c.131G>A, p.G44D	3.5	intramural	
	T3	c.131G>A, p.G44D	2.0	intramural	
	T4	c.110_118del9, p.T37_L39del	1.5	intramural	
My6104	T1	c.131G>A, p.G44D	2.0	submucous	
My6105	T1	wt	6.0	intramural	
	T2	wt	6.0	intramural	
	T3	wt	6.0	intramural	
	T4	wt	2.5	intramural	
	T5	wt	1.5	intramural	
My6106	T1	wt	3.0	intramural	
My6107	T1	wt	2.8	intramural	
My6108	T1	c.130G>A, p.G44S	5.0	submucous	
My6110	T1	wt	6.0	intramural	cellular leiomyoma
My6111	T1	wt	4.5	intramural	mitotically active leiomyoma
My6112	T1	wt	4.0	intramural	highly cellular leiomyoma
My6113	T1	c.131G>A, p.G44D	2.5	intramural	
	T2	c.131G>T, p.G44V	2.5	intramural	
	T3	c.130G>T, p.G44C	1.5	intramural	
	T4	c.123_152del30, p.K42_V51del	1.5	intramural	
	T5	c.100-8T>A, p.E33_D34insPQ	1.2	intramural	
My6114	T1	wt	1.1	intramural	
My6115	T1	c.131G>A, p.G44D	3.0	intramural	
	T2	c.131G>T, p.G44V	2.0	intramural	
My6116	T1	c.130G>A, p.G44S	2.5	intramural	
My6117	T1	c.131G>T, p.G44V	3.0	subserous	
	T2	c.131G>C, p.G44A	1.5	submucous	
	T3	c.131G>C, p.G44A	1.1	submucous	
My6118	T1	wt	18.0	intramural	
My6119	T1	c.131G>C, p.G44A	3.0	NA	
	T2	c.130G>T, p.G44C	2.0	intramural	
	T3	c.130G>A, p.G44S	1.5	intramural	
	T4	c.131_136del6, p.G44_N46delinsD	1.2	intramural	
My6120	T1	c.131G>C, p.G44A	8.0	intramural	
	T2	c.130G>C, p.G44R	2.5	intramural	
My6121	T1	wt	6.0	intramural	
	T2	c.130G>T, p.G44C	1.5	intramural	
	T3	c.100-62_141del104, loss of splice acceptor	3.0	intramural	
	T4	c.131G>A, p.G44D	2.0	intramural	
My6122	T1	c.130G>A, p.G44S	6.0	intramural	
	T2	c.107T>G, p.L36R	3.0	intramural	
	T3	c.130G>C, p.G44R	4.0	subserous	
	T4	c.131G>T, p.G44V	6.0	intramural	
	T5	c.83_99+5del22, p.D28_E33del	4.0	subserous	
	T6	c.130G>C, p.G44R	3.0	subserous	
	T7	c.130G>C, p.G44R	6.0	subserous	
	T8	c.100-8T>A, p.E33_D34insPQ	2.0	intramural	
	T9	c.131G>A, p.G44D	ND	NA	
My6123	T1	wt	6.0	intramural	

	T2	c.124_130del7insT, p.K42_G44delinsC	3.0	intramural	
	T3	c.108_122del15, p.T37_V41del	2.5	intramural	
	T4	c.131_136del6, p.G44_N46delinsD	2.0	intramural	
My6124	T1	wt	5.0	intramural	
	T2	c.125_130del6, p.K42_G44delinsS	3.0	intramural	
	T3	c.100_108del9, p.D34_L36del	1.3	intramural	
My6125	T1	wt	8.0	intramural	
	T2	wt	4.0	subserous	
My6126	T1	wt	10.0	intramural	
My6127	T1	c.130G>C, p.G44R	7.0	intramural	
	T2	c.117_137del21, p.L39_N46delinsF	5.0	submucous	
	T3	c.100-8T>A, p.E33_D34insPQ	3.0	subserous	
	T4	c.121_138del18, p.V41_N46del	7.0	intramural	
	T5	c.130G>A, p.G44S	3.0	intramural	
	T6	c.100-8T>A, p.E33_D34insPQ	3.5	intramural	
	T7	c.130G>A, p.G44S	4.0	intramural	
	T8	c.130G>T, p.G44C	4.0	subserous	
	T9	c.130G>C, p.G44R	4.0	subserous	
	T10	c.131G>A, p.G44D	3.0	submucous	
My6128	T1	c.131G>A, p.G44D	5.0	intramural	
My6129	T1	c.119_122del4insGAGA, p.N40_V41delinsRE	3.0	intramural	
	T2	c.131G>A, p.G44D	1.5	intramural	
	T3	c.131G>T, p.G44V	4.0	intramural	
My6130	T1	wt	9.0	intramural	
My6131	T1	wt	0.9	intramural	lipoleiomyoma
My6133	T1	wt	6.0	intramural	
My6134	T1	c.131G>A, p.G44D	ND	submucous	
	T2	c.130G>A, p.G44S	1.5	subserous	
	T3	c.128A>C, p.Q43P	1.5	submucous	
	T4	c.130G>C, p.G44R	1.7	intramural	
	T5	c.107T>G, p.L36R	4.0	intramural	
My6135	T1	c.131G>A, p.G44D	5.0	intramural	
	T2	c.100-8T>A, p.E33_D34insPQ	4.0	intramural	
	T3	wt	2.5	intramural	
	T4	wt	2.0	intramural	
My6136	T1	wt	4.0	intramural	
	T2	wt	2.3	intramural	
	T3	wt	2.0	intramural	
My6137	T1	c.133_150del18, p.F45_A50del	2.5	NA	
	T2	c.100-8T>A, p.E33_D34insPQ	2.5	NA	
	T3	c.141_164del24, p.N47_E55delinsK	3.0	intramural	
	T4	c.119_148del30, p.N40_A50delinsT	3.5	intramural	
	T5	c.130G>A, p.G44S	2.5	intramural	
	T6	c.100-8T>A, p.E33_D34insPQ	1.0	intramural	
My6138	T1	c.131G>T, p.G44V	2.5	intramural	
	T2	c.131G>C, p.G44A	2.0	submucous	
My6139	T1	wt	6.0	intramural	
	T2	c.131G>A, p.G44D	1.3	intramural	
	T3	c.131G>A, p.G44D	1.5	intramural	
	T4	c.130G>T, p.G44C	1.5	intramural	
	T5	c.131G>A, p.G44D	3.0	submucous	
My6140	T1	c.121_138del18, p.V41_N46del	4.5	NA	
	T2	c.131G>A, p.G44D	2.5	intramural	
	T3	c.130G>C, p.G44R	1.2	intramural	
	T4	c.130G>T, p.G44C	1.8	intramural	
	T5	c.131G>T, p.G44V	1.0	intramural	

	T6	c.130G>C, p.G44R	1.0	intramural
	T7	c.131G>A, p.G44D	2.5	intramural
	T8	c.131G>C, p.G44A	0.9	intramural
	T9	c.131G>T, p.G44V	0.8	intramural
	T10	c.130G>C, p.G44R	2.0	intramural
	T11	c.130G>A, p.G44S	2.0	intramural
	T12	c.130G>A, p.G44S	2.5	subserous
My6141	T1	c.128A>C, p.Q43P	8.0	NA
	T2	c.131G>A, p.G44D	8.0	NA
	T3	c.120_125del6, p.N40_V41del	6.0	NA
	T4	c.131G>A, p.G44D	6.0	subserous
	T5	c.130G>C, p.G44R	1.0	intramural
	T6	c.131G>T, p.G44V	1.0	intramural
	T7	wt	1.0	subserous
	T8	c.129_140del12, p.Q43_N47delinsH	1.0	intramural
	T9	c.130G>C, p.G44R	3.0	intramural
	T10	c.130G>C, p.G44R	2.5	intramural
My6142	T1	wt	7.0	intramural
	T2	wt	4.5	submucous
My6143	T1	c.107T>G, p.L36R	4.0	NA
	T2	c.130G>C, p.G44R	2.0	intramural
	T3	c.130G>C, p.G44R	2.0	intramural
My6144	T1	wt	5.0	intramural
	T2	wt	1.0	intramural
	T3	c.130G>C, p.G44R	0.5	intramural
	T4	wt	0.8	intramural
	T5	c.131G>A, p.G44D	0.8	intramural
My6145	T1	c.130G>T, p.G44C	5.0	intramural
	T2	c.107T>G, p.L36R	5.0	intramural
	T3	c.130G>T, p.G44C	5.0	intramural
	T4	c.130G>C, p.G44R	5.0	intramural
	T5	c.107T>G, p.L36R	8.0	intramural
	T6	c.131G>A, p.G44D	6.0	submucous
	T7	c.107T>G, p.L36R	4.0	intramural
	T8	c.107T>G, p.L36R	4.0	intramural
	T9	c.131G>A, p.G44D	5.0	intramural
	T10	c.131G>A, p.G44D	7.0	intramural
	T11	c.130G>T, p.G44C	5.0	intramural
	T12	c.131G>A, p.G44D	4.0	intramural
	T13	c.131G>C, p.G44A	3.0	intramural
	T14	c.130G>C, p.G44R	4.0	subserous
	T15	c.130G>A, p.G44S	3.0	subserous
My6146	T1	c.130G>T, p.G44C	4.0	intramural
	T2	c.130G>A, p.G44S	2.5	intramural
My6147	T1	c.131G>A, p.G44D	1.7	intramural
	T2	c.129_146del18, p.Q43_P49delinsH	0.8	intramural
	T3	c.131G>A, p.G44D	1.5	intramural
	T4	c.107T>G, p.L36R	1.2	intramural
My6148	T1	c.130G>A, p.G44S	5.0	intramural
	T2	c.130G>C, p.G44R	1.5	intramural
	T3	c.131G>T, p.G44V	7.0	NA
	T4	c.100-1_137del39, loss of splice acceptor	3.5	NA
	T5	c.131G>A, p.G44D	2.5	NA
My6149	T1	c.131G>A, p.G44D	5.0	intramural
My6151	T1	wt	5.0	NA
	T2	wt	4.0	intramural
My6152	T1	c.131G>A, p.G44D	6.0	intramural

	T2	c.130G>C, p.G44R	4.0	intramural
	T3	c.131G>A, p.G44D	5.0	intramural
	T4	c.131G>T, p.G44V	3.5	intramural
	T5	c.130G>A, p.G44S	3.0	intramural
	T6	c.100_135del36, p.D34_F45del	2.0	intramural
	T7	c.131G>A, p.G44D	2.0	intramural
	T8	c.131G>A, p.G44D	1.0	intramural
	T9	c.131G>A, p.G44D	1.0	intramural
My6153	T1	wt	7.0	intramural
My6155	T1	wt	9.0	intramural
My6156	T1	c.130G>A, p.G44S	4.5	intramural
	T2	c.131G>A, p.G44D	2.0	subserous
	T3	c.107T>G, p.L36R	2.0	intramural
My6158	T1	c.107T>G, p.L36R	2.0	intramural
	T2	c.130G>A, p.G44S	2.0	intramural
	T3	c.109_110ins9, p.L36_T37insMKL	0.8	intramural
	T4	c.130G>T, p.G44C	0.9	intramural
My6159	T1	c.128A>C, p.Q43P	1.4	intramural
	T2	c.131G>T, p.G44V	1.6	submucous
	T3	c.131G>A, p.G44D	7.5	intramural
My6160	T1	c.130G>A, p.G44S	5.0	submucous
	T2	c.131G>C, p.G44A	1.2	NA
	T3	c.130G>C, p.G44R	1.2	intramural
My6161	T1	c.130G>A, p.G44S	11.0	NA
	T2	c.131G>C, p.G44A	10.0	NA
	T3	c.115_141del27, p.L39_N47del	5.0	NA
	T4	c.102_128del27, p.D34_Q43delinsE	6.0	NA
	T5	c.131G>T, p.G44V	4.0	NA
	T6	c.107T>C, p.L36P	5.0	NA
	T7	c.131G>A, p.G44D	5.0	NA
	T8	c.102_128del27, p.D34_Q43delinsE	3.0	NA
	T9	c.130G>A, p.G44S	7.0	NA
	T10	c.130G>A, p.G44S	5.0	NA
	T11	c.108_122del15, p.T37_V41del	7.0	NA
	T12	c.130G>A, p.G44S	6.0	NA
	T13	c.128_130delAAG, p.Q43_G44delinsR	9.0	NA
	T14	c.146_166del21, p.P49_E55del	8.0	NA
	T15	c.128A>C, p.Q43P	2.5	NA
	T16	c.131G>T, p.G44V	2.0	NA
My6162	T1	c.131G>T, p.G44V	3.0	intramural
	T2	c.131G>A, p.G44D	0.7	intramural
My6163	T1	wt	9.0	intramural
	T2	wt	1.0	intramural
My6164	T1	wt	3.0	intramural
My6165	T1	c.131G>A, p.G44D	5.0	intramural
	T2	wt	1.0	intramural
My6166	T1	c.76_96del21, p.P26_K32del	2.5	subserous
	T2	c.129_150del22insG, p.G44_A50del	1.0	intramural
My6167	T1	wt	5.0	intramural
	T2	c.119_133del15, p.N40_F45delinsI	5.0	intramural
	T3	c.131G>T, p.G44V	2.5	intramural
	T4	c.130G>C, p.G44R	1.0	intramural
	T5	c.131G>A, p.G44D	1.0	intramural
	T6	c.131G>A, p.G44D	0.8	intramural
	T7	c.131G>A, p.G44D	2.0	intramural
My6168	T1	wt	3.0	submucous
	T2	c.107T>G, p.L36R	3.0	submucous

	T3	wt	1.5	intramural
	T4	c.131G>T, p.G44V	1.2	intramural
	T5	c.107T>G, p.L36R	0.9	intramural
	T6	c.131G>A, p.G44D	0.8	intramural
My6169	T1	c.130G>A, p.G44S	11.0	submucous
	T2	c.107T>G, p.L36R	6.0	intramural
	T3	c.130G>T, p.G44C	3.0	subserous
	T4	c.131G>A, p.G44D	5.0	subserous
	T5	c.130G>A, p.G44S	5.0	subserous
	T6	c.130G>C, p.G44R	1.5	intramural
My6170	T1	wt	2.5	intramural
	T2	wt	1.5	intramural
	T3	c.133_147del15, p.F45_P49del	0.9	intramural
My6171	T1	c.131G>A, p.G44D	4.0	intramural
	T2	wt	2.0	intramural
My6173	T1	wt	1.5	intramural
My6174	T1	wt	17.0	subserous
My6175	T1	wt	16.0	intramural
	T2	wt	10.0	intramural
My6176	T1	wt	17.0	intramural
My6177	T1	c.131G>A, p.G44D	14.0	intramural
My6178	T1	c.130G>A, p.G44S	5.0	subserous
	T2	c.131G>A, p.G44D	4.0	subserous
	T3	c.130G>T, p.G44C	4.0	intramural
	T4	c.130G>C, p.G44R	2.5	intramural
	T5	c.131G>A, p.G44D	1.5	subserous
	T6	c.131G>A, p.G44D	2.0	intramural
	T7	c.133_159del27, p.F45_G53del	1.5	subserous
	T8	c.131G>A, p.G44D	1.0	intramural
	T9	c.131G>A, p.G44D	1.5	subserous
	T10	c.130G>C, p.G44R	ND	NA
	T11	c.131G>T, p.G44V	3.5	intramural
	T12	c.100-6_131del39, loss of splice acceptor	2.5	intramural
	T13	c.130G>A, p.G44S	0.8	intramural
My6179	T1	c.149_163del15, p.A50_D54del	1.5	submucous
My6180	T1	c.130G>T, p.G44C	5.5	intramural
	T2	c.131G>A, p.G44D	5.0	intramural
	T3	c.130G>T, p.G44C	4.0	intramural
	T4	c.130G>A, p.G44S	4.0	submucous
	T5	c.100-2_107del10, loss of splice acceptor	2.5	intramural
	T6	c.131G>A, p.G44D	3.0	intramural
	T7	c.130G>T, p.G44C	3.0	intramural
	T8	c.130G>T, p.G44C	1.0	intramural
	T9	c.130G>C, p.G44R	9.0	intramural
	T10	c.131G>A, p.G44D	3.0	intramural
	T11	c.141_167del27, p.Q48_H56del	2.5	intramural
	T12	c.131G>T, p.G44V	1.5	intramural
	T13	c.100-8T>A, p.E33_D34insPQ	3.0	intramural
	T14	wt	1.0	intramural
	T15	c.131G>A, p.G44D	2.0	subserous
My6181	T1	c.131G>C, p.G44A	4.0	intramural
	T2	c.130_141del12, p.G44_N47del	1.0	intramural
	T3	c.107T>G, p.L36R	1.5	NA
My6182	T1	c.130G>T, p.G44C	13.0	intramural
	T2	c.130G>C, p.G44R	3.5	subserous
	T3	c.100-8T>A, p.E33_D34insPQ	3.0	intramural
	T4	c.122_127del6, p.V41_Q43delinsE	2.0	intramural

	T5	c.130G>C, p.G44R	1.5	intramural	
	T6	c.131G>A, p.G44D	1.5	intramural	
	T7	c.130G>T, p.G44C	1.0	subserous	
My6183	T1	c.131G>A, p.G44D	8.0	intramural	
	T2	c.131G>A, p.G44D	3.5	intramural	
	T3	c.131G>T, p.G44V	3.0	intramural	
	T4	c.130G>A, p.G44S	2.5	intramural	cellular leiomyoma
	T5	c.130G>T, p.G44C	2.5	intramural	
	T6	c.131G>A, p.G44D	2.0	intramural	
	T7	c.131G>C, p.G44A	2.0	intramural	
	T8	c.114_149del36, p.L39_A50del	1.5	intramural	
	T9	wt	3.5	intramural	leiomyoma with bizarre nuclei
My6184	T1	c.100-8T>A, p.E33_D34insPQ	5.0	NA	
	T2	c.131G>C, p.G44A	4.0	NA	mitotically active leiomyoma
	T3	c.100-8T>A, p.E33_D34insPQ	3.0	NA	epithelioid leiomyoma
My6185	T1	wt	8.0	intramural	
	T2	wt	2.0	intramural	
My6186	T1	wt	7.0	subserous	
	T2	wt	5.5	intramural	
	T3	wt	5.5	NA	
	T4	c.130G>C, p.G44R	3.5	NA	
My6187	T1	c.131G>A, p.G44D	2.5	intramural	
	T2	c.131G>A, p.G44D	3.5	intramural	
My6188	T1	c.130G>A, p.G44S	8.0	intramural	
My6189	T1	wt	13.0	intramural	
	T2	c.131G>T, p.G44V	1.5	intramural	leiomyoma with bizarre nuclei
	T3	wt	1.5	intramural	
My6190	T1	wt	11.0	intramural	
My6191	T1	wt	6.0	intramural	
	T2	c.131G>A, p.G44D	5.0	intramural	
	T3	wt	1.5	intramural	
My6192	T1	c.131G>C, p.G44A	7.0	intramural	
My6193	T1	wt	6.0	subserous	
My6194	T1	c.130G>T, p.G44C	9.0	intramural	
	T2	c.130G>C, p.G44R	2.5	intramural	
My6195	T1	wt	7.0	subserous	
My6196	T1	c.131G>A, p.G44D	5.0	subserous	
	T2	c.131G>A, p.G44D	2.0	intramural	
	T3	c.131G>A, p.G44D	0.6	intramural	
	T4	wt	1.0	intramural	
	T5	wt	1.0	intramural	
	T6	c.100-11_116del28,loss of splice acceptor	0.8	intramural	
My6197	T1	c.131G>A, p.G44D	ND	NA	
My6198	T1	c.131G>C, p.G44A	3.0	intramural	
	T2	c.131G>T, p.G44V	2.5	intramural	
	T3	c.131G>A, p.G44D	2.5	subserous	
	T4	c.131G>T, p.G44V	1.0	intramural	
	T5	c.130G>A, p.G44S	1.0	intramural	
My6199	T1	wt	7.0	intramural	
	T2	wt	4.0	intramural	
	T3	c.130G>A, p.G44S	2.0	intramural	
	T4	c.131G>A, p.G44D	1.0	intramural	
My6200	T1	c.131G>T, p.G44V	3.0	intramural	
	T2	c.131G>A, p.G44D	3.5	NA	
	T3	c.130G>T, p.G44C	6.0	intramural	

	T4	c.131G>A, p.G44D	2.0	intramural	
	T5	c.131G>A, p.G44D	1.0	intramural	
	T6	c.131G>C, p.G44A	1.0	intramural	
My6201	T1	c.131G>A, p.G44D	10.0	intramural	
	T2	c.79_93del15, p.Q27_Q31del	1.0	subserous	
	T3	c.130G>C, p.G44R	1.0	subserous	
My6202	T1	c.131G>A, p.G44D	8.0	subserous	
	T2	c.131G>T, p.G44V	3.0	intramural	
	T4	c.100-8T>A, p.E33_D34insPQ	2.0	intramural	
	T5	c.100-8T>A, p.E33_D34insPQ	1.5	submucous	
My6203	T1	wt	4.0	intramural	
	T2	c.130G>A, p.G44S	0.9	submucous	
My6205	T1	c.130G>A, p.G44S	2.0	intramural	
	T2	c.130G>T, p.G44C	2.5	intramural	
	T3	c.131G>A, p.G44D	6.0	intramural	
	T4	c.131G>A, p.G44D	4.0	intramural	
	T5	c.130G>A, p.G44S	2.0	intramural	
	T6	c.107T>G, p.L36R	2.0	intramural	
	T7	c.118_144del27, p.N40_Q48del	3.0	intramural	
My6206	T1	wt	4.0	intramural	highly cellular leiomyoma
	T2	c.130G>A, p.G44S	3.0	intramural	
	T3	c.134_150del17insGG, p.F45_A50delinsW	3.0	NA	
	T4	c.131G>A, p.G44D	1.0	intramural	
	T5	c.130G>A, p.G44S	1.0	intramural	
My6207	T1	c.107T>G, p.L36R	2.0	intramural	
	T2	c.130G>A, p.G44S	2.0	intramural	
	T3	c.130G>C, p.G44R	1.5	intramural	
	T4	c.131G>A, p.G44D	2.0	intramural	
	T5	c.131G>T, p.G44V	1.2	intramural	
My6208	T1	c.131G>T, p.G44V	5.5	intramural	
	T2	c.113_151del39, p.A38_A50del	3.5	intramural	
My6209	T1	wt	9.0	intramural	
My6210	T1	wt	6.0	intramural	
	T2	c.131G>A, p.G44D	4.0	intramural	
	T3	wt	3.5	intramural	
My6211	T1	c.131G>A, p.G44D	8.0	intramural	
	T2	wt	8.0	intramural	cellular leiomyoma
	T3	c.131G>A, p.G44D	3.5	intramural	
	T4	c.107T>G, p.L36R	3.0	intramural	
	T5	c.130G>C, p.G44R	6.0	intramural	
	T6	c.131G>A, p.G44D	3.5	intramural	cellular leiomyoma
	T7	c.117_134del18, p.L39_G44del	2.0	intramural	
	T8	c.109_144del36, p.T37_Q48del	1.0	intramural	
My6212	T1	c.123_134del12, p.K42_F45del	7.0	intramural	
	T2	c.107T>G, p.L36R	0.8	NA	
	T3	c.100-8T>A, p.E33_D34insPQ	0.8	NA	
	T4	c.83_99+1del18, p.D28_E33del	0.8	NA	
My6213	T1	wt	13.0	intramural	
	T2	c.131G>T, p.G44V	5.0	intramural	
	T3	c.131G>A, p.G44D	3.0	intramural	
My6214	T1	c.107T>G, p.L36R	ND	NA	
	T2	c.130G>C, p.G44R	1.0	intramural	
My6215	T1	wt	7.0	intramural	cellular leiomyoma
	T2	c.130G>C, p.G44R	1.0	intramural	
My6216	T1	c.100-21_151del73, loss of splice acceptor	3.5	intramural	
	T2	c.100-2_109dup12, p.L36_T37insKDEL	2.0	intramural	

	T3	c.131G>T, p.G44V	1.5	intramural	
	T4	c.131G>A, p.G44D	1.5	intramural	
	T5	c.130G>T, p.G44C	1.0	intramural	
	T6	c.130G>A, p.G44S	2.5	intramural	
	T7	c.130G>A, p.G44S	1.0	intramural	
	T8	c.123_134del12, p.K42_F45del	ND	intramural	
	T9	c.106_129del24, p.L36_Q43del	ND	intramural	
	T10	c.131G>T, p.G44V	5.0	intramural	
My6217	T1	wt	10.0	intramural	
	T2	c.132_158del27, p.F45_G53del	2.5	submucous	
	T3	c.130G>A, p.G44S	2.0	intramural	
	T4	c.131G>A, p.G44D	1.5	intramural	
My6218	T1	wt	6.0	NA	cellular leiomyoma
	T2	c.131G>T, p.G44V	3.0	intramural	
	T3	c.107T>G, p.L36R	2.0	intramural	
	T4	c.130G>A, p.G44S	1.8	intramural	
	T5	c.131G>A, p.G44D	1.0	intramural	
	T6	c.100-8T>A, p.E33_D34insPQ	0.8	subserous	
My6219	T1	c.107T>G, p.L36R	3.5	intramural	
My6220	T1	c.131G>T, p.G44V	4.0	intramural	
	T2	c.107T>G, p.L36R	2.5	intramural	
	T3	c.100-3_132del36, loss of splice acceptor	3.0	intramural	
	T4	c.131G>A, p.G44D	2.0	intramural	
My6221	T1	c.131G>A, p.G44D	5.0	intramural	
	T2	c.131G>A, p.G44D	2.0	intramural	
	T3	c.100-9_137del47, loss of splice acceptor	ND	intramural	
My6222	T1	c.107T>G, p.L36R	4.0	subserous	
	T2	c.100-8T>A, p.E33_D34insPQ	3.0	subserous	
	T3	c.126_146del21, p.K42_P49delinsN	3.5	subserous	
	T4	c.130G>T, p.G44C	4.0	subserous	
	T5	c.132_140del9, p.F45_N47del	3.0	intramural	
	T6	c.88_99del12, p.K30_E33del	1.0	intramural	
My6223	T1	wt	8.0	intramural	
	T2	wt	5.0	intramural	
	T3	wt	2.5	submucous	
	T4	c.131G>A, p.G44D	1.5	subserous	
	T5	c.130G>C, p.G44R	2.0	subserous	
	T6	c.133_144del12, p.F45_Q48del	3.0	intramural	
My6224	T1	c.131G>C, p.G44A	4.5	intramural	
	T2	c.131_139del9, p.G44_N47delinsD	1.5	intramural	
My6227	T1	wt	13.0	NA	
My6228	T1	wt	6.0	NA	
	T2	c.141_155del15, p.Q48_S52del	1.5	subserous	
My6229	T1	wt	4.0	intramural	
	T2	wt	2.0	intramural	
	T3	wt	1.2	intramural	
	T4	wt	2.5	intramural	
My6230	T1	c.131G>C, p.G44A	3.0	intramural	
My6231	T1	c.100-7_138del46, loss of splice acceptor	ND	NA	
	T2	c.130G>A, p.G44S	0.9	intramural	
	T3	c.107T>G, p.L36R	0.9	intramural	
My6232	T1	wt	ND	NA	
My6233	T1	c.130G>T, p.G44C	8.0	subserous	
	T2	c.130G>A, p.G44S	4.0	subserous	
	T3	c.131G>A, p.G44D	3.5	subserous	
	T4	c.131G>A, p.G44D	2.5	subserous	

	T5	c.118A>T(;)129_143del15, p.N40Y(;)G44_Q48del	1.5	subserous	
My6235	T1	c.100-2A>C(;)106_110del5ins100-30_100-12inv, p.D34_Q48del	6.0	subserous	
	T2	c.131G>A, p.G44D	8.0	intramural	
My6236	T1	c.131G>T, p.G44V	3.5	NA	
	T2	c.131G>T, p.G44V	4.0	intramural	
	T3	c.130G>A, p.G44S	3.0	intramural	
	T4	c.131G>A, p.G44D	2.0	intramural	
	T5	c.133_144del12, p.F45_Q48del	1.5	submucous	
	T6	c.130G>C, p.G44R	1.5	intramural	
	T7	c.100-4_143del48, loss of splice acceptor	2.0	intramural	
	T8	c.130G>C, p.G44R	2.0	intramural	
	T9	wt	1.5	intramural	
My6237	T1	c.131G>A, p.G44D	7.0	intramural	
	T2	c.131G>A, p.G44D	1.0	intramural	
My6238	T1	wt	5.0	intramural	lipoleiomyoma
My6239	T1	wt	2.0	intramural	
	T2	wt	1.5	intramural	
My6241	T1	c.131G>A, p.G44D	7.0	intramural	
	T2	c.130G>T, p.G44C	2.0	intramural	
	T3	c.130G>C, p.G44R	0.9	subserous	
My6242	T1	wt	6.0	NA	cellular leiomyoma
	T2	c.130G>T, p.G44C	1.5	intramural	
My6243	T1	wt	12.0	intramural	
	T2	wt	2.5	intramural	
	T3	c.131G>A, p.G44D	1.0	intramural	
My6244	T1	c.142_162del21, p.Q48_D54del	8.0	NA	
	T2	wt	3.0	intramural	
	T3	c.122T>A, p.V41E	2.0	intramural	
	T4	c.131G>T, p.G44V	1.5	intramural	
	T5	c.131G>T, p.G44V	1.0	intramural	
My6245	T1	c.130G>T, p.G44C	8.0	subserous	
	T2	wt	0.8	intramural	
My6246	T1	c.131G>A, p.G44D	4.0	subserous	
	T2	c.130G>C, p.G44R	2.0	intramural	
	T3	c.130G>C, p.G44R	1.0	intramural	
	T4	c.131G>T, p.G44V	1.0	intramural	
My6247	T1	c.130G>C, p.G44R	3.0	intramural	
	T2	c.129_149del21, p.Q43_A50delinsH	1.5	submucous	
	T3	c.130G>A, p.G44S	0.8	intramural	
	T4	c.131G>T, p.G44V	0.8	intramural	
	T5	c.110_118dup9, p.T37_L39dup	1.0	intramural	
My6248	T1	c.130G>A, p.G44S	8.0	intramural	
	T2	wt	7.0	intramural	
	T3	c.128A>C, p.Q43P	4.0	intramural	
My6249	T1	wt	2.0	intramural	
My6250	T1	wt	8.0	intramural	
My6251	T1	wt	8.0	intramural	
My6252	T1	wt	11.0	intramural	
My6253	T1	c.130G>T, p.G44C	7.0	intramural	
	T2	c.131G>A, p.G44D	6.5	intramural	
	T3	c.131G>A, p.G44D	5.0	intramural	
	T4	c.130G>C, p.G44R	2.0	intramural	
	T6	c.117_136del20insTC, p.L39_N46delinsFH	2.5	submucous	
	T7	c.100-8T>A, p.E33_D34insPQ	2.5	intramural	
	T8	c.130G>A, p.G44S	5.0	intramural	

	T9	c.131G>A, p.G44D	3.5	intramural	
	T10	c.107T>G, p.L36R	5.0	subserous	
	T11	c.121_138del18, p.V41_N46del	6.0	intramural	
	T12	c.100-8T>A, p.E33_D34insPQ	6.0	intramural	
	T13	c.130G>A, p.G44S	4.0	intramural	
	T14	c.131G>A, p.G44D	2.0	subserous	cellular and mitotically active leiomyoma
My6254	T1	wt	7.0	intramural	
My6255	T1	wt	6.0	subserous	
My6256	T1	c.107T>G, p.L36R	2.5	intramural	
	T2	wt	1.0	intramural	
	T3	c.128A>C, p.Q43P	2.0	intramural	
My6257	T1	c.128A>C, p.Q43P	2.5	intramural	
	T2	c.130G>T, p.G44C	4.0	subserous	
My6258	T1	c.130G>A, p.G44S	3.0	NA	
	T2	c.131G>A, p.G44D	1.0	intramural	
My6259	T1	c.100-8T>A, p.E33_D34insPQ	3.0	intramural	
My6260	T1	wt	8.0	intramural	
	T2	c.131G>C, p.G44A	1.5	subserous	
	T3	c.128A>C, p.Q43P	2.0	intramural	
	T4	c.130G>A, p.G44S	2.0	intramural	
	T5	c.130G>C, p.G44R	1.5	intramural	
	T6	c.131G>T, p.G44V	1.5	intramural	
	T7	c.146_166del21, p.P49_E55del	1.0	intramural	
	T8	c.100-8T>A, p.E33_D34insPQ	1.0	intramural	
	T9	c.[120_121insAA;120_121ins100-10_120], p.N40_V41insNLPQDELTALN	1.3	intramural	
My6261	T1	c.131G>A, p.G44D	6.0	intramural	
	T2	c.129_137del9, p.Q43_N46delinsH	3.5	NA	
	T3	c.131G>C, p.G44A	1.5	intramural	
	T4	c.122_139del18, p.V41_N47delinsD	2.0	intramural	
My6262	T1	c.131G>A, p.G44D	7.0	intramural	
	T2	c.130G>C, p.G44R	1.0	intramural	
My6263	T1	c.100-23_110del34, loss of splice acceptor	4.0	intramural	
My6264	T1	c.131G>T, p.G44V	4.5	intramural	
	T2	c.131G>A, p.G44D	2.5	submucous	
	T3	c.131G>A, p.G44D	1.0	intramural	
My6265	T1	c.130G>C, p.G44R	15.0	intramural	
	T2	c.106_129del24, p.L36_Q43del	2.5	intramural	
	T3	c.131G>C, p.G44A	2.0	intramural	
My6266	T1	c.130G>A, p.G44S	3.5	submucous	cellular leiomyoma
	T2	wt	0.8	intramural	
My6267	T1	wt	16.0	intramural	

Note: MED12-mutation status is presented as nucleotide change and predicted protein change. NA = not available; wt = wild type.
